# Transurethral ethanol ablation for symptomatic benign prostatic hyperplasia

**Published:** 2007

**Authors:** Gagan Gautam, Nitin Arora

**Affiliations:** Department of Urology and Renal Transplant, Fortis Flt. Lt. Rajan Dhall Hospital, Vasant Kunj, New Delhi, India. E-mail: gagangg@gmail.com

## SUMMARY

A prospective randomized multicenter trial was conducted to assess the safety and efficacy of transurethral ethanol ablation of the prostate (TEAP) as a treatment for men with symptomatic benign prostatic hyperplasia (BPH). Seventy-nine men with a mean (range) age of 64 (50-79) years, who had drug refractory voiding symptoms (International Prostate Symptom Score, IPSS > 12) and prostate volumes of 30-80cc as recorded by preoperative transrectal ultrasound (TRUS) were recruited. Patients with a predominant median lobe hyperplasia and those with a history of a surgical/minimally invasive procedure for BPH were excluded from the study. Ethanol was injected transurethrally into the lateral lobes of the prostate with a curved cystoscopic needle in men randomly assigned to one of the three doses: 15% (low dose), 25% (medium dose) or 40% (high dose) of prostate volume by TRUS. The mean volume of ethanol injected and the mean operating time was 14.0 ± 5.11 ml and 25 min respectively. Foley's catheter was placed after the procedure and was retained for a minimum of three days after which more than 98% of the subjects were able to void. On follow-up till six months, statistically significant improvements were seen in terms of the IPSS, quality of life (QoL) score, peak flow rate (PFR) and prostate volume reduction across all groups. In the low-dose group, IPSS, QoL score, PFR and prostatic volume improved from 22.7 ± 5.21, 4.5 ± 0.89, 8.3 ± 2.55 ml/min and 46.1 ± 15.36 cc to 12.1 ± 7.80, 2.2 ± 1.74, 11.5 ± 5.07 ml/min and 39.8 ± 16.20 cc respectively at six months follow-up. Similar improvements were also seen in the medium-dose (IPSS 24.4 ± 5.22 to 11.0 ± 8.61, QoL 4.4 ± 1.03 to 2.2 ± 1.63, PFR 8.1 ± 2.22 to 13.5 ± 6.57 ml/min, TRUS volume 44.3 ± 13.27 to 38.7 ± 18.32cc) and high-dose (IPSS 23.7 ± 5.03 to 11.2 ± 4.88, QoL 4.2 ± 0.89 to 2.1 ± 1.19, PFR 8.6 ± 2.11 to 16.69 ± 10.73 ml/min, TRUS volume 45.2 ± 12.67 to 34.0 ± 15.98 cc) treatment groups. These improvements were consistently observed across the three groups without an apparent dose effect. Adverse events were recorded at each visit and included hematuria (41.8%), irritative voiding symptoms (39.2%), pain/discomfort (27.8%), urinary retention (21.5%) and urinary incontinence (13.9%). Of these events 75% were reported shortly after the procedure, were mostly mild to moderate in severity and typically resolved within the first four weeks, including complaints of irritative voiding. One patient required a transurethral resection (TURP) for persistent obstructive voiding symptoms while another underwent cystoscopy to extract the broken tip of a cystoscopic needle one month after the primary procedure. Prostate volume and ethanol dose did not exert any influence on the incidence and type of adverse events in this study. The authors concluded that transurethral ethanol ablation of the prostate was safe and effective in the short term and held promise as a minimally invasive treatment modality for BPH in the future.

## COMMENTS

Although TURP continues to be the established modality for the treatment of drug refractory and complicated BPH, its association with potentially serious complications and morbidity has prompted the search for newer and minimally invasive therapies. Unfortunately, none of these new modalities (except perhaps, laser ablation) have been able to match up to the benefits achieved by TURP in terms of peak flow rates and reduction of symptoms.[1] Moreover, all of these (including laser) are associated with a greater cost of treatment, which may prevent their widespread use in our society.

TEAP is a new technique, which uses an easily available low-cost agent, can be performed under local anesthesia in an office setup and does not require any complicated equipment. Moreover, it has not been shown to be associated with any serious potential complication, which would preclude its use in a daycare setting.

This study has been able to replicate the findings of a recent European multicenter trial, which showed a significant improvement in lower urinary tract symptoms at the one-month follow-up visit after TEAP, which was maintained through 12 months follow-up. In this study, IPSS and QoL scores decreased by more than 50%. The PFR improved by 35% by the third month and the average prostate volume reduction was 16%. Adverse events included discomfort or irritative voiding symptoms in 26% patients, hematuria in 16% and retrograde ejaculation in less than 3% patients. The majority of these events required no intervention.[2] Other small pilot studies have also indicated the positive impact of this treatment on outcome parameters of symptomatic BPH.[3]

A potential cause of concern with this treatment is the relatively high incidence of irritative side-effects, urinary retention, pain and dysuria, which may decrease the acceptability of this procedure in some patients. Moreover, there is a prolonged period of catheterization (more than three days) used in this study protocol, which in fact is the same or even more than that after TURP. However, as further research is done with regards to the optimum dosage, delivery system, patient selection and follow-up protocols of TEAP, we can expect to see a dilution of some of these negative factors and the emergence of this treatment as a safe, effective and economical alternative to TURP.
